# Adherence to Medications after Hospital Discharge in the Elderly

**DOI:** 10.1155/2013/901845

**Published:** 2013-03-26

**Authors:** Elie Mulhem, David Lick, Jobin Varughese, Eithne Barton, Trevor Ripley, Joanna Haveman

**Affiliations:** Department of Family Medicine, Oakland University William Beaumont School of Medicine, Rochester, MI 48314, USA

## Abstract

*Objectives*. To evaluate the adherence rate to prescribed medications in elderly patients 24–48 hours after being discharged from the hospital. *Methods*. Family medicine residents visited patients over the age of 65 years at their homes one to two days after being discharged from the hospital and documented all the medications that they were taking since coming home from the hospital. The list of medications was later compared to the medications recorded in hospital discharge instructions. *Results*. Complete data was available for 46 participants. The average patient age was 76 years; 54.4% were women. Only three patients (6.5%) adhered completely to the discharge medication list found in the medical record. Thirty-six patients (78.2%) reported taking at least one additional prescription medication, twenty patients (43.4%) missed at least one prescription medication, twenty patients (43.4%) reported taking the wrong dose of at least one medication, and nineteen patients (41.3%) reported taking medications at an incorrect frequency. *Conclusion*. The vast majority of elderly patients in our study did not adhere to the medication regimen in the first two days after hospital discharge. Cost-effective improvements to hospital discharge processes are needed to improve adherence and reduce preventable posthospitalization complications.

## 1. Introduction

The elderly are at a significant risk for medication-related problems, including nonadherence, especially at times of transition in and out of the health care system. Many factors contribute to the risk of nonadherence in older patients including a higher prevalence of chronic diseases, a higher number of prescription and nonprescription medications compared to any other age group, and age-related physical and mental capabilities that also may pose a challenge to their ability to adhere to prescribed medications regimens [1]. It is well known that nonadherence to medications can result in worsening clinical outcomes, including rehospitalization, exacerbation of chronic medical conditions, increased healthcare costs, and death [2–4]. Robin Dimatteo et al. reported that the odds of poor health outcomes are nearly three times higher for patients who do not adhere to recommended therapies compared to patients who follow providers instructions [2].

The transition from hospital to home is a particularly vulnerable time for elderly patients, and nonadherence to prescribed medication regimen after discharge from the hospital increases the risk of postdischarge complications. Forster et al. reported that one in five hospitalizations is complicated by postdischarge adverse events, and 66% of these events are related to medications [5]. Additionally, 33–69% of medication-related hospital admissions in the United States are due to medication nonadherence with a resultant cost of approximately $100 billion per year [6].

Hospital admission typically results in changes to medication regimens [7]. Although these medication changes are communicated to the patient at the time of discharge, rates of adherence to the new medication regimen at home vary widely in studies [8–11]. While many hospitals have improved their medication reconciliation processes in the recent years, the literature is lacking in the evaluation of the short-term effectiveness of these changes on medication adherence after the patient goes home.

The purpose of this study was to document the level of adherence of elderly patients to the prescribed medication list shortly after discharge from the hospital. This was performed as a pilot study before implementing changes to the medication communication portion of the hospital discharge process.

## 2. Methods

Our study is a qualitative survey conducted at Beaumont Hospital, Troy, a community hospital in suburban southeast Michigan. We received approval for the study from our institution's Human Investigations Committee.

The current discharge process in our hospital includes the review of the written discharge medication list with the patient by the nurse who informs the patient and/or their family of new medications and discontinued medications from their home medication list. Changes to medications are often but not always reviewed with the patients and their families by the attending physician.

We identified patients 65 years of age or older who could be enrolled in the study from general surgical or medical units. These patients were identified from the daily list of patients that the hospital care managers maintained with expected discharge dates. Their hospital records were reviewed by members of the study team, and patients were disqualified from the study if they were being discharged to a facility other than their home, if they had a documented diagnosis such as dementia that would impair their cognition, or if they did not reside in the two surrounding counties. Patients were enrolled from May 2009 through January 2010.

After identifying patients that met the above criteria, family medicine residents met with each patient in the hospital and obtained informed consent to participate in the study. The investigator informed the patient that the study's goal was to evaluate how well patients are able to follow hospital discharge instructions and that the investigator visiting them at home would ask them about the medications they have been taking after they left the hospital. Patients were also informed that the investigator would not know what medications they should be taking nor would he/she be able to answer any questions about their medications. The patient's medical care providers were not informed that the patient was being included in a medication adherence study. At that time, an appointment's date and time were agreed upon for the interviewer to meet the patient at their home. This appointment was scheduled within 24–48 hours of discharge from the hospital, and the visit was conducted by the research personnel who obtained informed consent in the majority of cases.

At the home visit, the interviewer asked the patient to gather all the medications that they have been taking since discharge from the hospital, including prescription medications, over-the-counter medications, supplements, and vitamins. The patient was asked to name the medications they were taking and describe how they were taking them, including how many pills and how many times per day. We documented the medications, dosages, and frequency as shown and described by the patient. The collected data was recorded in a preprinted table at the time of the home interview and transferred to an Excel spread sheet later.

A second investigator recorded the list of medications that the patient was instructed to take at the time of discharge by a review of the medical record and instructions that were provided to the patient at the time of discharge from the hospital. This list was then compared to the list obtained at the home visit for data analysis. Both investigators were unaware of the other list at the time their respective list was compiled. Demographic factors such as age, sex, length of hospital stay, number of chronic diseases, and number of medications were also documented for analysis. Chronic diseases were defined as those medical conditions that were documented in the discharge summary, were present for at least three months prior to admission or were new diagnoses that were expected to be present for at least three months after discharge from the hospital.

## 3. Statistics

We used Spearman's rank correlation for pairs to compare each individual demographic factor to each type of medication error. We documented five patient demographics and six types of medication errors. The five demographics included patient age, gender, number of medications, length of stay in the hospital, and number of chronic diseases. The errors documented included addition and omission of prescription medications, addition and omission of over-the-counter medications, errors in frequency, and errors in the dose taken. A high positive correlation coefficient (approaching 1) indicates a strong correlation in the same direction, whereas a high negative correlation coefficient (approaching −1) indicates a strong correlation in the opposite directions. We used the Wilcoxon Rank Sum test to calculate *P* values for each of the pairings.

## 4. Results

**Figure 1:**
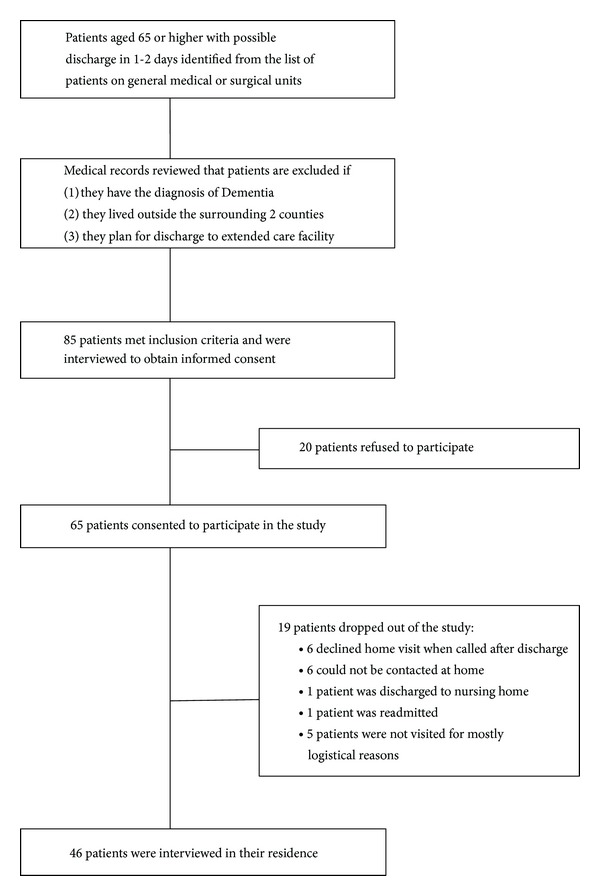
Study flow diagram.

We identified 85 patients that met the criteria for inclusion in the study. Twenty declined to participate in the study. Of the 65 patients who consented to participate, 19 patients were lost to followup resulting in 46 visits to patients at their residence. This is shown in Figure 1.

We did home visits to 25 (54.4%) women and 21 (45.6%) men. The average age of the patients interviewed was 76 years. The mean number of medications listed on the hospital discharge instructions was 9.98 prescription medications and 1.83 nonprescription medications per patient. Patients had an average hospital length of stay of 4.31 days and averaged 6.98 chronic diseases listed on their discharge summary. Patient demographics are shown in Table 1.

Based on patient-reported medication lists during the home interview, only three patients (6.5%) adhered completely to the discharge medication list documented in the discharge instructions. Thirty-six patients (78.2%) reported taking at least one additional prescription medication that was not found in the discharge instructions. The number of additional prescription medications ranged from one to fifteen additional medications. Twenty patients (43.4%) omitted at least one prescription medication that was ordered in the discharge instructions. The number of omitted medications ranged from one to six medications. Twenty patients (43.4%) reported taking the wrong dose of at least one medication with four patients taking as many as three medications incorrectly. Nineteen patients (41.3%) reported taking medications at a different frequency than prescribed with one patient taking as many as four medications incorrectly.

A total of 95 medication discrepancies were documented when compared to the discharge medication list, with adding medications being the most common discrepancy (37.8%). Medication omissions, dosing discrepancies, and frequency discrepancies occurred with similar frequency (19%-20%).

**Figure 2:**
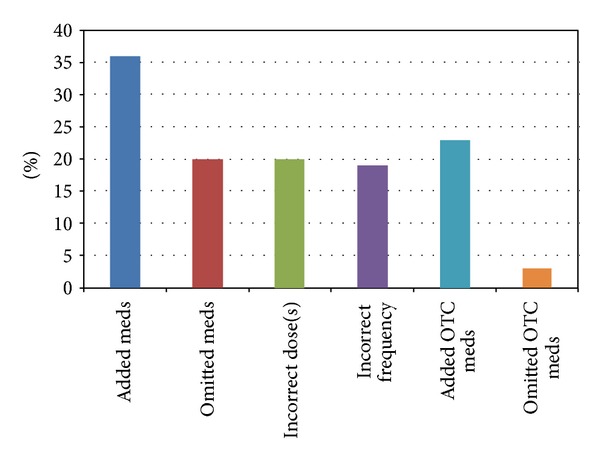
Distribution of patients with the four types of medication nonadherence for prescribed and over-the-counter (OTC) medications.

Twenty-three patients (50%) reported taking at least one nonprescription medication (over-the-counter medication, vitamin, or herbal supplement) that was not listed on their discharge instructions. The number of nonprescription medications added ranged from one to nine. Three patients (6.5%) omitted at least one nonprescription medication from their prescribed regimen with a range of one to three medications omitted. Patient compliance results are shown in Figure 2.

Table 2 shows the correlation results between each individual demographic factor and each type of medication error. The strongest associations are positive associations between the number of medications at discharge and the number of medication dosing and frequency discrepancies. These two are the only associations that achieve statistical significance. There were no statistically significant differences between men and women for each medication discrepancy type. All *P* values were greater than 0.46.

## 5. Discussion

It is well known that medication regimens frequently change over the course of a hospitalization [7, 12, 13]. The vast majority of elderly patients in our study did not adhere to the medication regimen documented in their discharge instructions in the first two days after hospital discharge. The most common discrepancy occurred when patients reported taking medications that were not recommended at the time of hospital discharge (78%), followed by omitting at least one medication (43%).

In a similar study published in 1992, Beers et al. evaluated adherence using elderly patients self-reporting two days after hospitalization. They found that 64% of patients added medications and 50% missed at least one medication. Although the care of patients has changed significantly in the past twenty years, this problem of medication nonadherence seems to continue with little change. One important factor contributing to these discrepancies is that patients do not always recall all home medications when they are admitted to the hospital, and they restart these medications when they are back home. This causes a discrepancy between the prescribed discharge medication list and what the patient is actually taking at home. While this is an important issue, this does not reflects a true nonadherence to the patient's home medications.

Over-the-counter medications pose another challenge in maintaining accurate medication lists and discharge instructions. Many medication classes, such as pain relievers, laxatives, and acid reducers, are available over-the-counter and may also not be reported by the patient at the time of hospitalization.

We did find a strong positive association between the number of mediations taken by the patient and the risk of discrepancy in dose or frequency. Other studies also reported a decrease in adherence with an increase in the number of medications taken and an increase in the number of daily doses [14–16]. We also found evidence of a positive association between dosing discrepancies and frequency discrepancies (patients who had more dosing discrepancies tend to make more frequency discrepancies and vice versa).

The main limitation of our study is the small sample size of patients. However, this study was intended as a pilot study to be used as a benchmark for future interventions in our hospital. Another limitation is that we measured adherence based on patients self-reporting of the medications they were taking but we did not know if the patients reported all of these medications at time of admission to the hospital. Although patients' self-reporting is susceptible to errors, it is considered to be an effective method of measuring adherence [6, 17].

Various trials have evaluated different interventions to decrease drug-related problems in the elderly after hospital discharge, ranging from followup from a dedicated pharmacist to major and costly changes to the hospital discharge processes. These interventions have had varying effects on medication adherence and postdischarge quality measures, but few cost-effective strategies have been described [18, 19].

We are using these results to work collaboratively with pharmacists, nurses, hospital administrators, and elderly patients to address this multifactorial problem in a cost-effective way. Our future intervention to improve medication adherence will include first establishing an accurate home medication list by calling the patient's pharmacy before the patient is discharged home and reconciling the pharmacy medication list with the list given by the patient at time of hospital admission. This should help create a more accurate home medication list since we found that the home medication list is frequently incomplete when it is recalled by the patient. The second aspect of our intervention is to use a new user-friendly medication calendar to communicate discharge medication. We created this calendar by combining the Mayo Clinic discharge medication calendar with other calendars used to communicate medication regimens to patients with low health literacy [20, 21]. The third aspect includes an educational video that will be viewed by patients at time of discharge. This video will inform patients about the importance of adherence to medications, common causes of nonadherence, and its negative effects on their health. The video will also teach patients how to use the new medication calendar and how to reconcile medication at home. Finally, a pharmacy hot line will be made available for patients to call when they encounter questions about their medications.

Adherence to the prescribed discharge medication list is still very low in elderly patients despite an increased emphasis in newer discharge processes. There continues to be a significant need for innovative and cost-effective processes that improve compliance in this vulnerable and growing population.

## Figures and Tables

**Table 1:** Patient demographics.

Variables	Mean (range)
Age	76 years (65–94)
Gender	
Male	21 (45.6%)
Female	25 (54.4%)
Number of medications per patient	9.98 prescription meds (2–21) 1.83 herbal/vitamins (0–9)
Length of stay	4.31 days (1–13)
Number of chronic diseases	6.98 diseases (2–14)

**Table 2:** Spearman's rank correlation for patient demographics and types of medication discrepancies.

Medication discrepancy	Age	Length of stay	Number of diagnoses	Number of discharge medications
Patients taking additional prescription medications	−.089	−.158	.061	.249
Patients omitting prescription medications	−0.46	−.166	−.015	−.209
Patients taking the wrong dose	−.074	.144	.046	.481
Patients taking the wrong frequency	.021	.182	.253	.477
Patients taking additional nonprescription medication	−.223	−.082	−.043	.252
Patients omitting nonprescription medication	−.007	−.108	.094	.399
